# Application of data linkage techniques to Pacific Northwest commercial fishing injury and fatality data

**DOI:** 10.1186/s40621-021-00323-z

**Published:** 2021-07-05

**Authors:** Jasmine Nahorniak, Viktor Bovbjerg, Samantha Case, Laurel Kincl

**Affiliations:** 1 College of Earth, Ocean, and Atmospheric Sciences, Oregon State University, 104 CEOAS Admin Bldg., Corvallis, OR 97331 USA; 2 College of Public Health and Human Sciences, Oregon State University, 160 SW 26th St., Corvallis, OR 97331 USA; 3 National Institute for Occupational Safety and Health, Centers for Disease Control and Prevention, Suite 310, 4230 University Drive, Anchorage, AK 99508 USA

**Keywords:** Fishing, Vessel, Safety, Injury, Fatality, Linkage

## Abstract

**Background:**

Commercial fishing consistently has among the highest workforce injury and fatality rates in the United States. Data related to commercial fishing incidents are routinely collected by multiple organizations which do not currently coordinate or automatically link data. Each data set has the potential to generate a more complete picture to inform prevention efforts. Our objective was to examine the utility of using statistical data linkage methods to link commercial fishing incident data when personally identifiable information is not available.

**Methods:**

In this feasibility study, we identified true matches and discrepancies between de-identified data sets using the Python Record Linkage Toolkit. Four commercial fishing data sets from Oregon and Washington were linked: the Commercial Fishing Incident Database, the Vessel Casualty Database, the Nonfatal Injuries Database, and the Oregon Trauma Registry. The data sets each covered different date ranges within 2000–2017, containing 458, 524, 184, and 11 cases respectively. Several data linkage classifiers were evaluated.

**Results:**

The Naïve-Bayes classifier returned the highest number of true matches between these small data sets. A total of 41 true matches and 8 close matches were identified, of which 29 were determined to be duplicates. In addition, linkage highlighted 4 records that were not commercial fishing cases from Oregon and Washington. The optimum match parameters were the date, state, vessel official number, and number of people on board.

**Conclusions:**

Statistical data linkage enables accurate, routine matching for small de-identified injury and fatality data sets such as those in commercial fishing. It provides information needed to improve the accuracy of existing data records. It also enables expanding and sharpening details of individual incidents in support of occupational safety research.

## Background

Commercial fishing consistently has one of the highest workforce injury/fatality rates in the United States (Lucas & Case, 2018; Bureau of Labor Statistics, 2018). To gain a clear understanding of the hazards and opportunities for incident and injury prevention in the commercial fishing industry, it is critical to have a more complete picture of injury characteristics and burden in this workforce. Effective safety strategies can be informed by knowledge of the circumstances leading up to the incident, actions taken to prevent or minimize injury, resulting injuries or fatalities, and injury treatment and outcomes.

US data related to commercial fishing incidents are routinely collected by multiple organizations. Linking these data sets is not straightforward. Each data set has strict restrictions on data use, unique data request procedures and costs, different inclusion criteria, different data collection and maintenance methods, varying update rates and data lags, different storage formats, and differing data elements and data definitions. Safety-related data sets also often come with multiple challenges: small numbers of incidents, inconsistent values across data sets, and missing data. In addition, personally identifiable information, such as the victim’s name, is secured by the source and usually not available for matching. Evaluating the level of difficulty of obtaining routine access to and working with these data sets is a critical component of the overall project under which this linking feasibility study was completed.

Statistical data linkage techniques exist that not only help identify true matches in these cases, but also provide statistics describing the linkage confidence. The utility of statistical data linkage for linking de-identified data has been successfully demonstrated in other health-related fields (Conderino et al., 2017). A recent study used a combination of data linkage software and manual verification to link Alaskan commercial fishing data with moderate success (Syron, 2021).

Here, we assess an alternative method to provide routine, accurate, automated data linkage of commercial fishing data. The method is applied to data from Oregon and Washington, two states with active commercial fisheries on the west coast of the United States. While this study focused on commercial fishing incident data from the Pacific Northwest, the statistical matching methods presented here could be easily adapted to other regions, industries and occupations.

## Methods

Two data sets at a time were linked to determine their overlap. Data linkage software was used to automatically suggest potential matches by comparing the link probabilities to a threshold, then confirmed manually. This process was repeated for all permutations of the four data sets (a total of six pairs). The following sections describe the data sets and linkage approach.

### Data sets

The following data sets with commercial fishing incident data from Oregon and Washington were studied: the Commercial Fishing Incident Database, the Vessel Casualty database, the Nonfatal Injuries database, and the Oregon Trauma Registry.

The Commercial Fishing Incident Database (CFID) contains information regarding commercial fishing vessel disasters and fatalities from the entire United States (CDC/NIOSH, 2019). The CFID definition of a vessel disaster is an event such as sinking that forces the crew to abandon the vessel because it is no longer safe to remain onboard. The types of data collected include the date, time, and location of the incident, vessel details, contributing factors, and personnel injury and fatality information. A single incident may involve injuries to and/or fatalities of multiple personnel. The original sources of data used to populate this database include United States Coast Guard (USCG) reports and news articles. CFID was developed and is actively maintained by the Centers for Disease Control and Prevention’s National Institute for Occupational Safety and Health (NIOSH), Western States Division.

The Vessel Casualty database recorded information about commercial fishing vessel-related incidents in Alaska, Oregon, and Washington that (a) are not classified as vessel disasters and (b) did not involve any fatalities. These incidents tend to be less serious but still present problems with vessel systems that can put crewmembers at risk, such as loss of power, propulsion, or steering (Case & Lucas, 2020). This data set was maintained by NIOSH and merged with CFID in 2020. Cases were originally obtained from USCG reports. This data set provides information about the incident date, time, location, and circumstances, in addition to the vessel information. Unlike CFID, this data set does not include any personnel information, focusing instead on vessel damage. For this study, data were requested from Oregon and Washington only.

The Nonfatal Injuries database was developed to complement the information recorded in CFID. To date, this data set has covered Alaska, Washington, Oregon, and California. The Nonfatal Injuries database records injuries sustained during commercial fishing that are not included in CFID, such as those incurred while working on deck. Cases were originally obtained from USCG reports. Variables in this data set are similar to those in CFID; in addition to personnel demographics and injury characteristics, the Nonfatal Injuries database also contains vessel information. Data were requested from Oregon, Washington, and California only.

The Oregon Trauma Registry (Oregon Health Authority Public Health Division, 2019) includes information concerning all Oregon patients who either entered into the trauma system in Oregon or met specific clinical- or admission-based criteria for inclusion in the registry, based either on field entry by EMS responders or by the activation of a trauma team or surgeon at a receiving hospital. Information recorded includes patient demographics (including occupation), date, time and location of incident, emergency service response, injury circumstances and details, medical procedures performed, length of stay, insurance, and costs. Data were requested for patients with work-related injuries and occupations of farming/fishing/forestry. This data set was further pruned using incident location and narratives to include only fishing-related cases. The Oregon Trauma Registry is maintained by the Injury and Violence Prevention Program of the Oregon Health Authority Public Health Division.

Each data set used in this study contains data from different date ranges and regions (Table 1). The date range varied by data source due to lags in data abstraction, coding, and review, or to availability of data elements during specific time periods.

**Table 1:** Number of commercial fishing incidents recorded in each of the data sets used in this study

Data Set	Date Range (YYYY-MM-DD)	Region	Total ^a^	OR/WA ^a^
Commercial Fishing Incident Database	2000-01-04 to 2017-12-04	All USA	1315 (2966)	194 (458)
Vessel Casualty	2010-08-15 to 2014-12-31	OR/WA	524 (0)	524 (0)
Nonfatal Injuries	2002-01-12 to 2016-10-19	OR/WA/CA	232 (232)	184 (184)
Oregon Trauma Registry	2009-05-08 to 2016-07-06	OR	11 (11)	11 (11)

^a^ Each incident may involve multiple personnel. The number between parentheses is the total number of personnel cases.

**Fig. 1:**
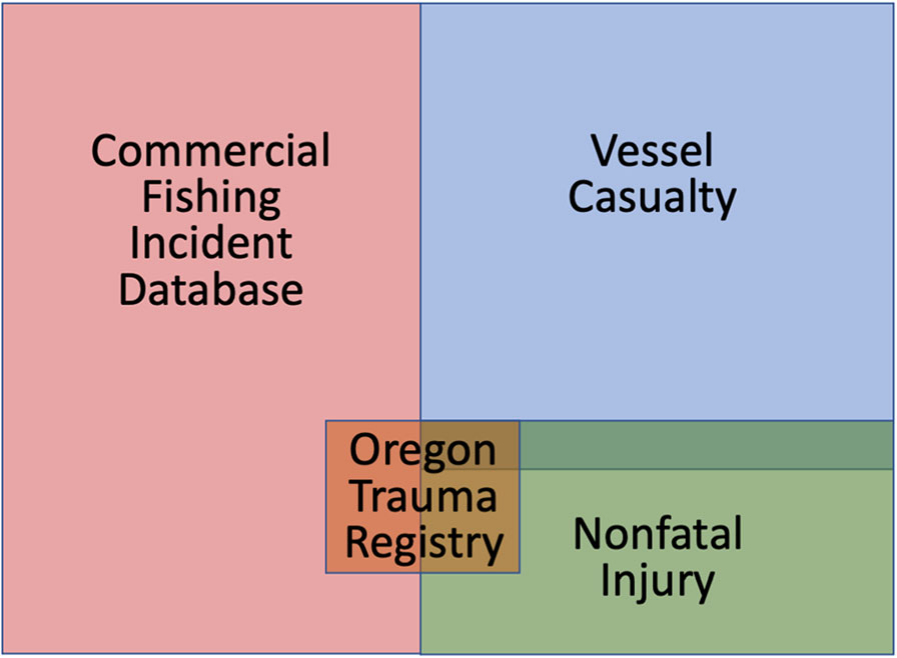
Schematic (not to scale) of the expected overlap between commercial fishing incidents in the following data sets: Commercial Fishing Incident Database, Vessel Casualty, Nonfatal Injuries, and the Oregon Trauma Registry

By definition, (a) the Commercial Fishing Incident Database contents should not overlap the Vessel Casualty or Nonfatal Injuries data sets, (b) the Vessel Casualty and Nonfatal Injuries data sets may overlap, and (c) the Oregon Trauma Registry data set could overlap with any of them (Fig. 1).

### Data linkage method

Data linkage is a statistical technique used to identify records from two data sets that likely describe the same event. The two data sets must have some parameters in common (i.e., the matching variables) that can be used to distinguish events and link the records. Every record in one data set is compared with every record in a second data set. The likelihood that two records match (their match probability) is determined by comparing the contents of the matching variables for that pair. Match probabilities range from 0 to 1. Any record pair with a match probability above a specified threshold is designated as a link. Those below the threshold are designated as non-links. In our project, this was followed by a manual review process where all links were examined further to identify true matches.

Matching variables must be selected carefully. Ideal matching variables are independent, reliable, and complete. A major aim of this project is to determine the feasibility of using data linkage methods with commercial fishing incident data when personally identifiable information (PII) is not available. Depending on the two data sets involved, the matching variables used for linking in this study were some combination of: Incident Date, Incident State, Vessel Official Number, and Latitude/Longitude. These independent variables were identified during preliminary data linkage analyses to be the strongest indicators of links.

The linking results presented here were derived using components of the Python Record Linkage Toolkit software (De Bruin, 2019). This toolkit includes several data linkage classifiers, which use different methods to separate record pairs into links and non-links. The quality of the performance of each classifier depends on the data set involved. Each of the classifiers described below were tested to determine the optimum classifier for our data sets.

Classifiers can be divided into two groups: supervised and unsupervised. Supervised classifiers require training using a “golden data set”, a subset of the data where the true match status is known. Unsupervised classifiers, on the other hand, do not require training.

For the supervised classifiers, a golden data set was derived for each pair of data sets to be linked. First, a rudimentary approach was used to identify a small list of potential matches. True matches were then verified manually. Next, a set of non-matches was derived by creating fake records from scrambled real records. Finally, a golden data set for the data set pair was created consisting of a combination of these verified true- and non-matches. For this study, the supervised classifiers used were Naïve-Bayes, logistic regression, and support vector machine.

The unsupervised classifier used in this study was the Expectation/Conditional Maximization Algorithm.

### Classifier definitions

The Naïve-Bayes classifier is a supervised classifier closely related to a foundational probabilistic approach developed by Fellegi and Sunter in 1969 (Fellegi and Sunter, 1969). This classifier assumes that the match parameters are (a) independent and (b) of equal weight. During training, this classifier examines each parameter from the “golden data set” and calculates the probability that a specific parameter value contributes to a match (or not). The resulting lookup table of weights is then used to determine the probability that two new item sets are a match. Two item sets are considered a match if their calculated match probability is greater than their non-match probability (i.e., match probability > 0.5).

Logistic regression is a deterministic, supervised classifier. It is used only in cases where the dependent (output, predicted) variable is binary (e.g., match/non-match, 0/1). Logistic regression derives parameters that describe the regression, allowing predictions. In addition to outputting the predicted value (0 fail, 1 success), this classifier also provides the odds that the variable takes the predicted value. The coefficients of the logistic regression algorithm are derived using maximum likelihood estimation. In logistic regression, probabilities are mapped between 0 and 1 using a Sigmoid function (an S-shaped curve, also called the logistic function). (Note that the values of this function never quite reach 0 or 1).

The support vector machine classifier is a supervised classifier that utilizes machine learning. This algorithm works by assigning the training data to points in space, such that data from the two categories (link or non-link) are separated by a large gap. It then derives the optimal hyperplane that separates these two clusters of points. New data are then classified depending on which side of the plane it falls on. While support vector machine classifiers exist that are able to derive non-linear hyperplanes, the only version available through the Python Data Linkage Toolkit is linear. It is a non-probabilistic, binary classifier. A disadvantage of this classifier is that it does not provide any estimates of the confidence of the derived link/non-link status. It only outputs 0 (non-match) or 1 (match), hence this classifier requires high confidence in the data.

The Expectation/Conditional Maximization Algorithm is a probabilistic classifier closely related to both the Naïve-Bayes classifier and the probabilistic Fellegi and Sunter (1969) approach. The Expectation/Conditional Maximization Algorithm (developed by Meng and Rubin, 1993) is an extension of the Expectation-Maximization algorithm (Dempster et al., 1977). This classifier assumes that the match parameters are independent. This classifier is used to find local maximum likelihood estimates of parameters when data are incomplete. To do so, it takes an iterative approach to solving simultaneous equations of the derivatives of the likelihood functions. It begins by making a random guess at model parameters, then gradually tweaks them until a zero derivative is found. A drawback of this approach is that it may return a local maximum (or saddle) instead of the global maximum.

### Quality metrics

Quality metrics provided by data linkage include TP (the number of True Positives), FP (False Positives), FN (False Negatives), and TN (True Negatives). For supervised classifiers, these describe how well the linkage technique performed on the golden data set. Additional metrics can be derived including precision, recall and f-score (Eqns. 1–3, respectively). Each of these three metrics (precision, recall, f-score) can range in value from 0 (worst) to 1 (best).

1 $$ precision=\frac{true\ positives}{total\ predicted\ positives}=\frac{TP}{TP+ FP}\kern1em $$

2 $$ recall=\frac{true\ positives}{total\ actual\ positives}=\frac{TP}{TP+ FN} $$

3 $$ f- score=2\ast \frac{precision\ast recall}{precision+ recall}\kern7.1em $$

The f-score is a summary metric of the classifier’s performance that balances precision and recall. Generally, a higher f-score indicates better performance.

## Results

**Fig. 2:**
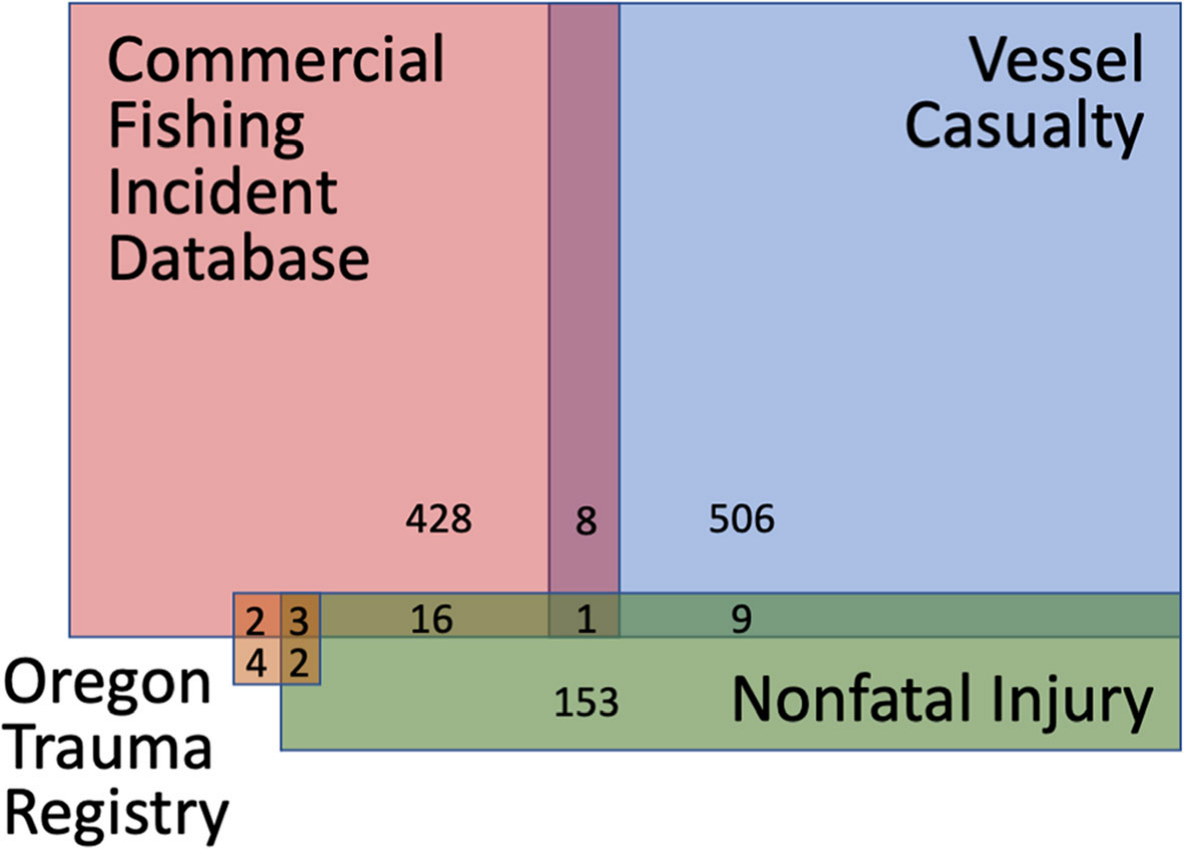
Schematic (not to scale) illustrating the number of true and close matches found between commercial fishing incidents in the following data sets: Commercial Fishing Incident Database, Vessel Casualty, Nonfatal Injuries, and the Oregon Trauma Registry

Data linkages were assessed for all combinations of the four data sets (a total of six pairs), regardless of whether matches were expected, to determine the efficacy of the method. In addition, the data linkage process was repeated for each of the four classifiers, for a total of 24 runs. The data linkage quality metrics are summarized in Table 2. No matches were found between the Vessel Casualty data set and the Oregon Trauma Registry. After manual review, a total of 41 true matches plus 8 close matches were found (Table 3). Four cases overlapped three data sets (Table 3, Fig. 2).

**Table 2:** Linkage metrics TP (true positives), FP (false positives), FN (false negatives), TN (true negatives), and f-score for each classifier per pair of data sets

**Commercial Fishing Incident Database & Oregon Trauma Registry** Match Parameters: Incident Date, Incident State Combinations (2966 * 11): 32,626 Golden Matches: 5						
**Classifier**	**Threshold**	**TP**	**FP**	**FN**	**TN**	**f-score**
Expectation/Conditional Maximization	0.5	4	6	1	32,615	0.53
Support vector machine	0.5	0	0	5	32,621	0
Naïve-Bayes	0.005	5	29	0	32,592	0.26
Logistic regression	0.005	5	29	0	32,592	0.26
**Commercial Fishing Incident Database & Vessel Casualty** Match Parameters: Incident Date, Vessel Official Number, Latitude/Longitude Combinations (1315 * 524): 689,060 Golden Matches: 9						
**Classifier**	**Threshold**	**TP**	**FP**	**FN**	**TN**	**f-score**
Expectation/Conditional Maximization	0.5	9	3	0	689,048	0.86
Support vector machine	0.5	8	0	1	689,051	0.94
Naïve-Bayes	0.005	9	3	0	689,048	0.86
Logistic regression	0.005	9	7	0	689,044	0.72
**Commercial Fishing Incident Database & Nonfatal Injuries** Match Parameters: Incident Date, Vessel Official Number, Latitude/Longitude Combinations (2966 * 232): 688,112 Golden Matches: 12						
**Classifier**	**Threshold**	**TP**	**FP**	**FN**	**TN**	**f-score**
Expectation/Conditional Maximization	0.5	12	52	0	688,048	0.32
Support vector machine	0.5	0	0	12	688,100	0
Naïve-Bayes	0.005	12	52	0	688,048	0.32
Logistic regression	0.005	12	52	0	688,048	0.32
**Nonfatal Injuries & Vessel Casualty** Match Parameters: Incident Date, Vessel Official Number, Latitude/Longitude Combinations (232 * 524): 121,568 Golden Matches: 10						
**Classifier**	**Threshold**	**TP**	**FP**	**FN**	**TN**	**f-score**
Expectation/Conditional Maximization	0.5	10	13	0	121,545	0.61
Support vector machine	0.5	9	1	1	121,557	0.90
Naïve-Bayes	0.01	10	2	0	121,556	0.91
Logistic regression	0.01	10	13	0	121,545	0.61
**Nonfatal Injuries & Oregon Trauma Registry** Match Parameters: Incident Date, Incident State Combinations (232 * 11): 2552 Golden Matches: 4						
**Classifier**	**Threshold**	**TP**	**FP**	**FN**	**TN**	**f-score**
Expectation/Conditional Maximization	0.2	4	3	0	2545	0.73
Support vector machine	0.5	0	0	4	2548	0
Naïve-Bayes	0.005	4	3	0	2545	0.73
Logistic regression	0.005	4	7	0	2541	0.53
**Vessel Casualty & Oregon Trauma Registry** Match Parameters: Incident Date, Incident State Combinations (524 * 11): 5764 Golden Matches: 0

**Table 3:** Number of true matches found for each data set combination

	Commercial Fishing Incident Database	Oregon Trauma Registry	Vessel Casualty	Nonfatal Injuries	Matches ^a^
Data Set Pairs	x	x			5
	x		x		9
	x			x	12 (20)
		x	x		0
		x		x	5
			x	x	10
				***Total***	**41 (49)**
Multiple Data Sets	x	x	x		0
	x	x		x	3
	x		x	x	1
		x	x	x	0
	x	x	x	x	0
				***Total***	**4**

^a^ The numbers in parentheses include close matches

**Fig. 3:**
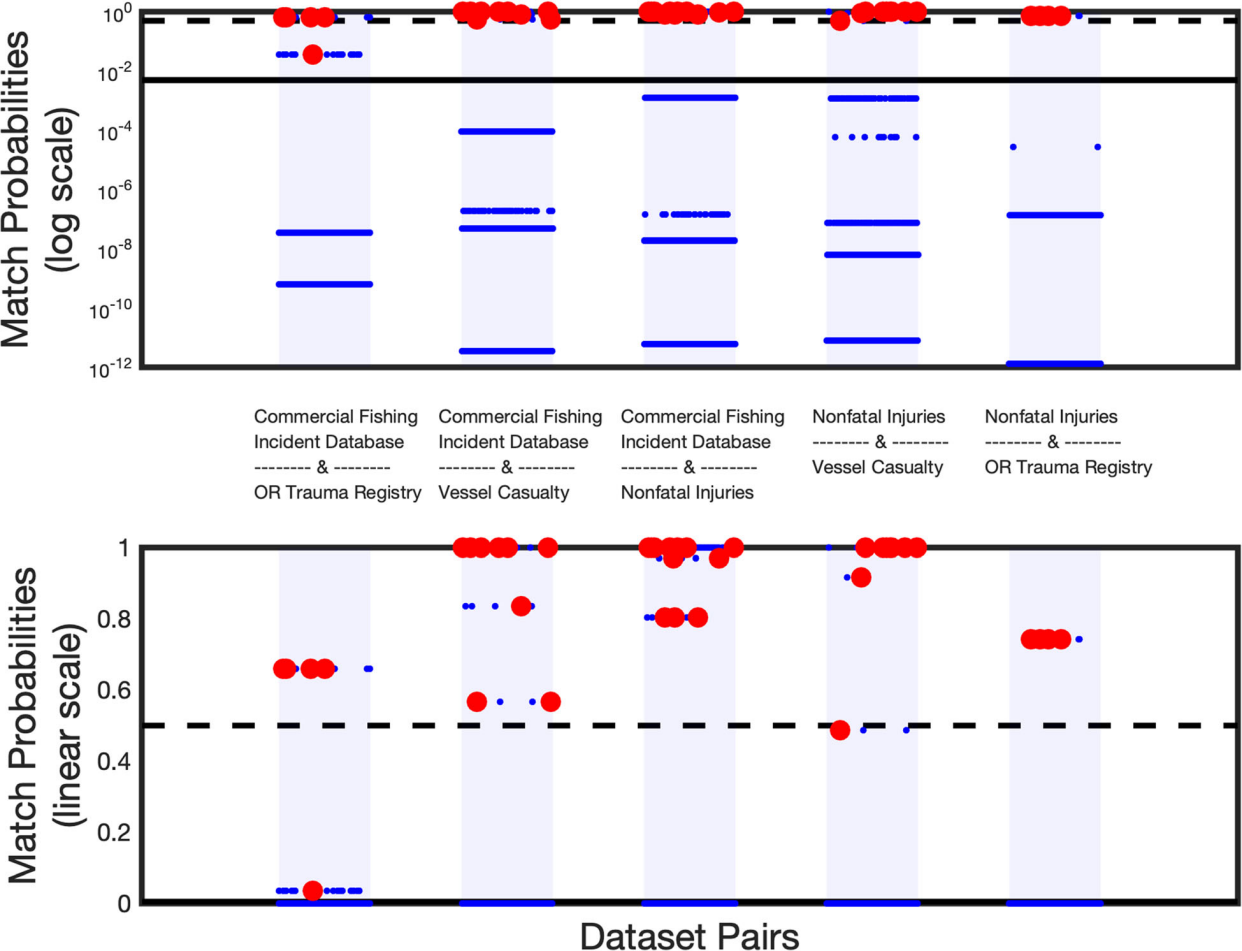
Match probabilities from the Naïve-Bayes classifier for all possible links found across different data set pairs. The threshold of 0.005 is shown as a solid line. A probability of 0.5 is indicated by a dashed line. True matches are highlighted with red circles. The same information is plotted on a log scale (upper panel) and linear scale (lower panel)

Three of the classifiers (Naïve-Bayes, Expectation/Conditional Maximization Algorithm, and logistic regression) provide match probability values for each possible link. The probabilities range from 0 (not a match) to 1 (exact match) and indicate the confidence that the pair is a true match. The probabilities output from the Naïve-Bayes classifier for every possible link in the pairs of data sets are shown in Fig. 3.

For the 41 true matches, the values of some common parameters were compared to assess the relative accuracy and completeness of the records (Table 4). Note that not all parameters listed in the table are provided by all data sets.

**Table 4:** Relative accuracy and completeness of data within the 41 true match pairs

Parameter	Relative Accuracy	Completeness	Commercial Fishing Incident Database^a^	Vessel Casualty^a^	Nonfatal Injuries^a^	Oregon Trauma Registry^a^
Incident Date	+/− 1 day	complete	x	x	x	x
Incident Time	varied from complete to no agreement	AM/PM designation and time zone often missing	x	x	x	x
Incident State	always agreed	complete	x	x	x	x
Latitude/Longitude	+/− 0.5 degrees (50 km)	often missing from trauma registry	x	x	x	x
Miles from Shore	never agreed	complete	x	x	x	
Vessel Official Number	always agreed	sometimes unavailable; state number used instead	x	x	x	
# People on Board	always agreed	only occasionally missing	x	x	x	
Narrative	consistent stories; matching provides additional details	rarely missing	x	x	x	x

^a^ The rightmost columns indicate the data sets that provide the listed parameters

## Discussion

Overlapping information from multiple sources (true matches) can serve multiple purposes. For data set pairs where overlaps are expected, the matching rows can be compared for accuracy and merged to obtain a more complete story. For data set pairs where no overlaps are expected yet overlaps are discovered, the overlap highlights possible errors or duplicates. And finally, for two data sets where overlaps between them are expected but not found, novel cases can be identified. Thus, each data set has the potential to identify new incidents, assess data accuracy, or provide complementary data to generate a more complete picture.

True matches and discrepancies between four commercial fishing data sets were successfully identified using the Python Record Linkage toolkit with different classifiers. The match results highlight the relative accuracy of parameters common to these data sets.

The small sizes of the commercial fishing data sets, ranging from 11 to 1316 cases, provides both advantages and challenges. The small number of incidents, spread over many years, means that Incident Date is a strong matching variable (the likelihood of two incidents occurring on the same day is small). On the flip side, the small data sets make it difficult to create a representative golden data set for the supervised classifiers.

Due to the small number of cases, it is important to capture every match. Hence, the optimal data linkage approach errs on returning false positives rather than false negatives. Ideally (a) all of the matches previously identified by the golden data set are discovered by the linkage tool, which implies also that (b) no false negatives are returned. In addition, it is helpful if the absolute number of false positives returned is low to limit the amount of manual review required to identify true matches.

Four classifiers were evaluated (Expectation/Conditional Maximization Algorithm, Naïve-Bayes, logistic regression, and support vector machine) for each combination of data sets. The classifier that was the least successful at identifying matches for these small data sets was support vector machine. The binary nature of this classifier meant that the threshold cannot be changed from 0.5, so improvement of these results was not possible. The Expectation/Conditional Maximization Algorithm classifier, on the other hand, performed very well; only one match was missed using a threshold of 0.5. The strongest performers were Naïve-Bayes and logistic regression, both of which successfully found all true positives but only after lowering the thresholds to 0.01 or 0.005. The optimum classifier was Naïve-Bayes which returned an additional true match between the Nonfatal Injuries data set and the Oregon Trauma Registry that was not found by any of the other classifiers. This particular match was difficult to find as the incident state information was missing.

Typically, a threshold of 0.5 is used to separate possible matches from non-matches. However, it can be seen from Fig. 3 that a much lower threshold (0.005) was necessary to capture all of the true matches in these particular data sets. This speaks to the inherent data variability both within and between the data sets. For example, while date was found to be a relatively reliable field, the date of the incident and the date of entry into a trauma registry may not always coincide due to the remoteness of the work.

Of the four data sets, the Oregon Trauma Registry was the most challenging to link. While this registry identifies the industry and occupation of injury cases, the latitude and longitude fields are often blank and vessel information is not recorded. This limited matching variables to Incident Date and Incident State.

The low number of records in the Oregon Trauma Registry (11) could indicate that (a) the number of commercial fishing injuries that were classified as trauma was extremely low, (b) not all commercial fishing related traumas were captured by the trauma registry variables “work-related” and “farming / fishing / logging”, and/or (c) some traumatic injuries did not make it into the trauma registry. To our knowledge, no data or research has been conducted that assesses the comprehensiveness of occupational coding or the completeness for trauma injury coverage at this time, but should be considered for future research.

These Oregon Trauma Registry results and conclusions may not be representative of other state trauma registries. Trauma registry data sets differ from state to state in their format, variables captured, access policies, and data request procedures. This is also true of many other sources of occupational health and safety data. Accommodating and accounting for this variety is a significant challenge to studies that span multiple states.

Another challenge occurs when multiple personnel are involved in a single incident. In this situation, a single record in one data set may link correctly with multiple records in another data set if the matching variables do not allow individual personnel to be distinguished. This results in false positives. For example, if three personnel are injured in a single incident and are all recorded in both the Commercial Fishing Incident Database and the Nonfatal Injuries database, it may not be possible to identify true patient-level matches with certainty unless the records contain enough information to distinguish them. This study identified eight injury cases that could not be matched with certainty down to the person level; they were designated as close matches (Table 3).

Data linkage is also useful for identifying extraneous records. For example, if overlaps are found between data sets that should be distinct, the results can be used to purge the data set(s) of the errant records. The Commercial Fishing Incident Database stores vessel disasters and fatalities while the Vessel Casualty database stores nonfatal vessel casualties. By definition, there should be no overlap between these two data sets, however nine true matches were found. These particular cases were unintentionally recorded twice; once in the Commercial Fishing Incident Database classified as vessel disasters and again in the Vessel Casualty database classified as vessel casualties. This highlighted a potential issue in the vessel disaster classification process which was then corrected.

Similarly, there should be no overlap between the Commercial Fishing Incident Database and the Nonfatal Injuries data set, however 12 true matches and an additional 8 close matches were found. The close matches involved incidents where multiple personnel were involved and could not be distinguished. All 20 incidents involved vessel disasters and/or fatal incidents and hence they should have only been recorded in the Commercial Fishing Incident Database and not the Nonfatal Injuries database. In addition to the previous example, this illustrates the utility of data linkage in the data cleaning process.

After accounting for the duplicates, 16 true matches remain out of the original 49. Five are matches between the Oregon Trauma Registry and the Commercial Fishing Incident Database, two are matches between the Oregon Trauma Registry and the Nonfatal Injuries data set, and nine are matches between the Nonfatal Injuries and Vessel Casualty data sets. Since each data source provides information about different aspects of an incident, these matches provide a wealth of additional information about each case that would otherwise be unavailable.

Valuable information can also be gleaned from the absence of expected true matches. For example, four of the 11 Oregon Trauma Registry cases were not found in any of the other commercial fishing incident data sets, prompting closer examination. Two of the four cases had an incident location of Crescent City, California, which is outside of the area covered by the other data sets. Based on their narratives, the remaining two cases may have involved recreational fishing rather than commercial fishing; the Commercial Fishing Incident Database, the Vessel Casualty database, and the Nonfatal Injuries database do not record recreational fishing incidents. Hence, non-matches with these four Oregon Trauma Registry cases are justified.

Arguably the greatest challenge in the analysis of commercial fishing injury and fatality data is unrecorded incidents. While vessel disasters and fatalities are well-captured via mandated USCG reporting requirements (Notice of marine casualty, 2019), nonfatal injuries are likely to be under-reported. This may result from concerns such as medical cost, future employment, fear of reprimand, insurance impact, and liability (Pransky et al., 1999). Vessel owners/operators may also be unaware of the mandatory casualty reporting requirements; a Maine study found that more than 40% of commercial fishing vessels were out of compliance with regulatory safety requirements (Davis, 2011). Trauma registries are also incomplete sources of injury information; only those cases that meet specific clinical- or admission-based criteria, based either on field entry by EMS responders or by the activation of a trauma team or surgeon at a receiving hospital, are included.

## Conclusions

Effective safety measures depend on accurate and complete information about potential hazards. Data linkage is a valuable tool that enables information from various sources to be merged, potentially yielding a more detailed picture of incidents from inception to outcome. In this study, four de-identified commercial fishing data sets were successfully linked using the Python Record Linkage Toolkit. Various classifiers were tested; the optimum classifier for this particular study was found to be the Naïve-Bayes classifier. A total of 41 true matches and 8 close matches were identified.

Data linkage also provides a means to assess the relative accuracy of common parameters. Of the parameters examined, the most reliable across the commercial fishing data sets were Incident Date, Incident State, Vessel Official Number, and the Number of People on Board. Knowledge of parameter reliability is essential for guiding appropriate matching variable choices for future data linkage analyses.

This effort is currently being expanded to include other geographic areas along the West Coast and additional data sources. This approach could further be tailored to a national level for commercial fishing, and/or to other occupational injury settings.

The outcomes of this study, the true matches, are also being assessed to better understand the injury causes, contributors, and outcomes to help inform prevention efforts.

## Data Availability

This study brought together existing data obtained upon request from a number of different sources. These data sets contain sensitive information and are not publicly available. These data may be requested from the original data providers (CDC/NIOSH and the Oregon Trauma Registry).

## Abbreviations

CDC: Centers for Disease Control and Prevention
CFID: Commercial Fishing Incident Database
EMS: Emergency Medical Services
FN: False negatives
FP: False positives
IRB: Institutional Review Board
NIOSH: National Institute for Occupational Safety and Health
PII: Personally identifiable information
TN: True negatives
TP: True positives
USCG: United States Coast Guard
